# Impacts of Autonomous Vehicles on Greenhouse Gas Emissions—Positive or Negative?

**DOI:** 10.3390/ijerph18115567

**Published:** 2021-05-23

**Authors:** Moneim Massar, Imran Reza, Syed Masiur Rahman, Sheikh Muhammad Habib Abdullah, Arshad Jamal, Fahad Saleh Al-Ismail

**Affiliations:** 1 Department of Civil & Environmental Engineering, College of Engineering and Applied Engineering, King Fahd University of Petroleum & Minerals, Dhahran 31261, Saudi Arabia; g201705990@kfupm.edu.sa (M.M.); ireza@kfupm.edu.sa (I.R.); arshad.jamal@kfupm.edu.sa (A.J.); 2 Center for Environment & Water, Research Institute, King Fahd University of Petroleum & Minerals, Dhahran 31261, Saudi Arabia; fsalismail@kfupm.edu.sa; 3 Department of Civil Engineering, Bangladesh University of Engineering & Technology, Dhaka 1000, Bangladesh; habibce09@gmail.com; 4 K.A. CARE Energy Research and Innovation Center (ERIC), King Fahd University of Petroleum & Minerals, Dhahran 31261, Saudi Arabia; 5 Department of Electrical Engineering, King Fahd University of Petroleum & Minerals, Dhahran 31261, Saudi Arabia

**Keywords:** autonomous vehicle, GHG, emission, COVID-19, CLD, energy consumption, VMT

## Abstract

The potential effects of autonomous vehicles (AVs) on greenhouse gas (GHG) emissions are uncertain, although numerous studies have been conducted to evaluate the impact. This paper aims to synthesize and review all the literature regarding the topic in a systematic manner to eliminate the bias and provide an overall insight, while incorporating some statistical analysis to provide an interval estimate of these studies. This paper addressed the effect of the positive and negative impacts reported in the literature in two categories of AVs: partial automation and full automation. The positive impacts represented in AVs’ possibility to reduce GHG emission can be attributed to some factors, including eco-driving, eco traffic signal, platooning, and less hunting for parking. The increase in vehicle mile travel (VMT) due to (i) modal shift to AVs by captive passengers, including elderly and disabled people and (ii) easier travel compared to other modes will contribute to raising the GHG emissions. The result shows that eco-driving and platooning have the most significant contribution to reducing GHG emissions by 35%. On the other side, easier travel and faster travel significantly contribute to the increase of GHG emissions by 41.24%. Study findings reveal that the positive emission changes may not be realized at a lower AV penetration rate, where the maximum emission reduction might take place within 60–80% of AV penetration into the network.

## 1. Introduction

According to the United Nations Framework on Climate Change Convention, the transportation sector was responsible for 27% of US greenhouse gas (GHG) emissions in 2010 [1]. GHGs are one of the leading causes of the greenhouse effect worldwide [2]. They serve as artificial heat-trapping agents within the earth’s atmosphere. From the perspective of road transportation, fuel sources such as diesel, natural gas, and gasoline produce different GHGs in the form of byproducts. Gaseous emissions resulting from burning these energy sources include methane (CH_4_), carbon dioxide (CO_2_) and nitrous oxide (N_2_O), which can last in the planet’s atmosphere for several decades, causing continuous global warming [3]. These unregulated GHGs emissions disturb the natural gas cycles governing the planet and pose a significant threat to various flora and fauna types [4]. In European countries, the transport sector was responsible for 30.5% of GHG emissions and 12% contribution of GHG emissions from road transport in 2014 [5]. Another study conducted in China by Liu et al. predicted that the transport sector alone would account for 84.7% GHG emission by the year 2040 [6]. Rising concerns about the negative environmental externalities of road transportation activity and development have urged governments worldwide to assess transportation projects’ environmental impacts before implementation. The modern automobile industry trend is to move towards the development of autonomous cars [7]. Multiple considerations are driving this change, including but not limited to improved safety, greater productivity, less fuel consumption and reduced traffic congestion [8,9]. Autonomous vehicles (AVs), also known as driverless or self-driving vehicles, are those vehicles that can operate without driver control the steering, accelerate or brake; the automation ranges from 0: no automation to 5: fully automated [10]. 

Existing literature on connected and autonomous vehicles mostly addresses their potential impact on the likelihood of traffic safety, travel behavior and congestion, as well as energy use. The effects of partially to fully automated vehicles on traffic performance and greenhouse gas emissions are still obscure. There are many uncertainties prevailing around the actual operation of fully automated vehicles. The Information Handling Services (IHS) Automotive experts reported that it is expected to happen by 2030. HIS estimates also suggest that globally the number of fully automated vehicles (AVs) in operation will be around 21 million in 2035 [11]. Another study reported that connected vehicles would strike the 250 million mark by 2020 [12]; a quarter of a billion cars in operation. A previous study also predicted that fully AVs be offered for auction before 2020 [13]. A projection is that AVs will dominate 20–40% of vehicle market share by 2030; however, it is believed that full-scale transition to AVs is likely to happen in stages over the coming few decades [14]. 

AVs are mainly equipped with contemporary car technologies, allowing computers to help in various driving operations and reduce human involvement to varying degrees. With rapid advances in communication, autonomous, and car technologies that have far-reaching effects on the transportation sector, it is critical to understand these technologies’ role in achieving sustainable urban mobility goals. This involves the safe and smooth operation of people and goods movement in an environmentally friendly manner. The carbon emission rate from each transport mode is significantly influenced by an array of factors, like the type of fuel, vehicle type, and age, etc. Many studies investigated the impacts of the widespread adoption of AV technology [15,16]. The impacts considered air pollutants, including GHG emissions. AVs’ introduction may contribute to increased ridesharing, traffic flow smoothing, platooning, efficient driving, efficient routing, eco traffic signal, and less hunting for parking [17,18,19,20,21]. As a result, the energy consumption will be less, contributing to the reduction of GHG emissions. A number of previous studies have investigated the role of AVs in improving transport sustainability by compressing energy use and GHG emissions. For example, one such estimation for the full automation developed by Wadud et al. considering the shared-vehicle scenario was based on the “Strong Responses” [22]. According to this concept, the maximum energy savings through car-sharing, eco-driving, right-sizing, and platooning are wholly neutralized by maximum energy increases from new user groups and higher speeds. In their study, Greenblatt and Shaheen explored the GHG reduction benefits of driverless taxis in the US and claimed that the deployment of each such taxi in the country would cause than 87–94% fewer emissions per vehicle-km trip by the year 2030 [23]. The authors also stated that each deployed driverless taxi in the same year would also cause a 63–82% reduction in GHG emissions than traditional fuel-driven and hybrid electric vehicles. Such reduction would primarily result from variations in three aspects: higher vehicle-km/vehicle/per-year increased fuel efficiency due to re-designed lighter/smaller vehicle sizes, less air friction, and reductions in GHG emissions through electricity consumption. On the other hand, AV may generate increased trips due to faster and more comfortable driving and new trips by captive passengers, such as elderly and disabled individuals [24]. 

Tomás et al. investigated the GHG implications of three different AV penetration rates (10, 20, and 30%) along an urban freeway corridor in the city of Porto, Portugal [25]. Authors used vehicle-specific power (VSP) and EEA-33 (environmental emergencies member countries) methodologies coupled with the VISSIM traffic model. It was noted that AVs yielded statistically low emission benefits at the corridor level at penetration rates less than 30%. In their study, Stasinopoulos et al. adopted a system dynamics approach and developed a stock and flow model to examine the GHG impacts of vehicle automation in various scenarios [26]. The study reported that emissions benefits of the transition to AVs might be negated by the inefficient use of AVs and induced demand. In another study, Wang et al. compared the fuel-cycle GHG emissions of AVs and vehicle electrification using an activity-based travel demand model for the Hamilton and Greater area [27]. It was concluded that full-scale induction of AVs would result in higher vehicle kilometers traveled, and hence, more GHG emissions are expected (2.5%). On the other hand, vehicle electrification may reduce vehicle emission intensities by approximately 11% and regional GHG emissions by over 5%. Hong and Zimmerman predicted that AVs can reduce GHG emissions by 20% compared to no-AV conditions in the year 2040, even under the worst-case scenario if vehicle automation provoked increased personal use with 85% vehicle fleet electrification [28]. A study conducted by Liu et al. also suggested that high AVs penetration rates in the long-term (by the year 2045) under optimistic scenarios will lead to a net reduction of GHG emissions [29]. 

This paper develops a landscape of multi-faceted issues related to GHG emissions from AV adoption at different levels by reviewing, synthesizing, analyzing, and comparing contrast research studies. While comparing the GHG emissions from AVs to its counterpart, fossil fuel vehicles (FFV) may have different attribute levels (e.g., gasoline-powered, eclectic, hydrogen-powered), this review study is only limited to the realm that both AVs and FFVs are only operated on fossil fuels. The study provides a causality analysis of GHG emissions from AVs from a holistic point of view. The primary objective of using a causal loop diagram (CLD) in our study is to understand the factors that can critically affect how the adoption of AVs may bring energy and GHG emission benefits to the transportation sector. CLD is used to see how these factors interact and influence the emission benefits of adopting AVs in the transport industry. Another section addressed the dynamics of GHG emissions during a global pandemic, focusing on travel behavior and how the individual vehicle ownership model may change in favor of adopting AVs. 

The remainder of this paper is structured as below. Section 2 provides an overview of the study methodology. Section 3 presents a description of the causes of GHG reduction by AVs, while the possible causes of the increase of GHG emission by adopting AVs are discussed in Section 4. Section 5 illustrates the changes in GHG emission at different AV penetration levels. Section 6 covers a discussion of the relationship between energy consumption and GHG emission; two sub-sections of Section 6 shed light on the causal loops of GHG emission from AVs from a system perspective and changed travel behavior during a global pandemic, respectively. Finally, Section 7 summarizes the study findings with concluding remarks.

## 2. Methodology

The systematic review has a formal protocol describing the strategy proposed for conducting the examination, identifying questions and methods employed to carry out the analysis [30]. The review process used in this study comprises three steps:

1. Planning: Defining the research issue, setting the criteria, identifying the limitation and development of the overall protocol.
2. Execution: Selection of research in database, categorizing useful references and bibliography, abstract of published manuscript.
3. Analysis: Summarizing the selected articles and classifying it to fit the proposed protocol.

**Figure 1:**
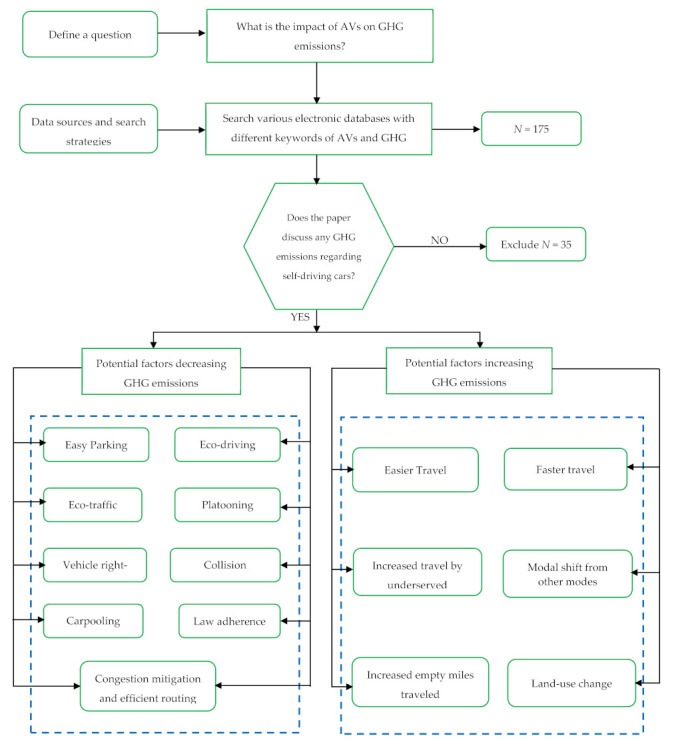
Methodology plan.

Various guidelines could manifest a systematic literature review. One of the popular methods is demonstrated by Kitchenham and Charters, a process that entails a number of tasks, including establishing a review protocol, identifying and selecting primary studies, extracting and synthesizing data, and finally, reporting study findings [31]. This paper focused on a systematic keyword search in the topic section of literature databases from disparate sources and repositories. The articles were searched for based on specific terms such as “autonomous vehicles,”; “self-driving car,” and “driverless car” appeared in the title, keywords, and abstract in the journal database. However, care was taken to single out the articles which were not focused on autonomous driving related to extensive applications, testing, and research in robotics, underwater vehicles, unmanned aerial vehicles, etc. The effects of AV-generated GHG emissions are explicitly investigated to achieve an overall classification to identify current gaps in the scientific literature in the realm of AV-related publications for roads, traffic studies related to commuting. The year of publication timeline and number of citations were taken out of the equation in selecting the articles to maximize the number for consideration. Articles found in different databases were also identified for eliminating duplication. The flowchart presented (Figure 1) illustrates the methodology deployed in this study. 

## 3. Causes of Reduction in GHG Emissions

This section provides a brief explanation of potential factors that are expected to reduce lower GHG emissions due to vehicle automation. Two types of vehicle automation strategies are considered, i.e., partial automation and full automation. 

### 3.1. Easy Parking

Guccione and Holland identified that drivers looking for parking are responsible for about one-third of traffic in the city [32]. From the fuel efficiency point of view, a vehicle searching for parking leads to a double threat. Being on the road consumes extra fuel for itself; the additional traffic makes the other vehicle suffer by staying more on-road and ending up using undue fuel. Roadside parking maneuver also has an important share in cities carbon emission system [33]. Shoup added to the literature with an estimation of 2–11% of total emission in a CBD being caused by parking hunt [34]. Easy parking refers to parking spaces’ availability through communication technologies that allow vehicles and infrastructure to exchange information, resulting in accurate parking information. In another study, Brown et al. estimated up to 5% of emissions in an average passenger car is attributed to the search for parking. Fully automated vehicles can achieve a 5–11% emission reduction from reduced circulation for parking in the cities [35]. Moriarty and Wang also estimated that parking space could be drastically reduced, and vehicles searching for parking could be cut down by 80% with shared ownership of AVs [10]. During peak traffic hours when congestion is high and off-peak travel periods, when most parking spaces may be occupied, the same reduction may occur. Partially automated vehicles would also minimize emissions due to improved ability to locate available parking spaces correctly; however, the projected savings could be lower, considering the lack of automatic implementation. In general, the easy parking feature of vehicle automation is expected to reduce GHG emissions depending upon various other factors, due to minimum vehicle idling and searching for suitable parking locations. 

### 3.2. Eco-Driving

Eco-driving refers to efficient driving through maximizing speed and acceleration operating profiles. Eco-driving is often referred to as “Hypermiling,” and is nothing but a set of driving skills practiced by enthusiastic drivers to push the fuel economy’s limit by minimizing braking-acceleration cycles, as braking causes a waste of energy [15,36]. CAV technologies have the ability to leverage and extend such efficient driving benefits by enabling vehicles to incorporate eco-driving automatically. CAVs can coordinate with other vehicles with smarter communication capability to make integrated driving decisions that would optimize overall traffic flow conditions and support the entire driving platoon. Barth and Boriboonsoms deployed a traffic simulation model to determine the emission effects of coordinated eco-driving [15]. The coordinated eco-driving system takes advantage of a virtual traffic management center to monitor vehicles’ speed and acceleration characteristics. They simulated a mixed fleet of vehicles on Southern California highways and estimated that carbon dioxide emissions reduction within a range of 10–20% could be achieved by eco-driving on congested highways. However, it has been noted that the reduction of emission starts to disappear as traffic approaches free flow. In a similar study, Barth demonstrated that a coordinated eco-driving system would minimize emissions by 5–10% in heavily congested road traffic [15]. Li and Gao conducted a series of micro-simulation modeling studies to investigate speed synchronization impacts in a connected environment [37]. Their primary objective was to establish an optimal control strategy to optimize fleet-level average fuel economy in a connected vehicle environment. The findings suggested that reducing 10% of GHG emissions could be achieved in such an arrangement. 

Two research projects conducted at the Virginia Tech Transportation Institute estimated potential emissions impacts of vehicle-to-vehicle (V2V) communication and coordination [19,38]. The proposed method involved complex optimization models integrating road-characteristics, information of the lead vehicle, vehicle acceleration portfolio, and microscopic fuel consumption models to produce a fuel optimal speed profile for vehicles in the network. Optimal driving cycles may reduce energy consumption by 35–50% under oversaturated conditions if these conditions exist at all in reality [39]. It is well known that frequent stops and accelerations/decelerations operations contribute to significant fuel consumption. The eco-driving attribute of AVs facilitates smooth vehicle navigation through the network, due to smart communication with other vehicles, as well as highway infrastructure, which in turn lowers the GHG emissions. 

### 3.3. Eco Traffic Signal

AVs can communicate with infrastructure on their own, particularly with traffic signals at intersections. This communication offers information to vehicles, which helps them change their driving pattern, thereby minimizing the number of stops at the intersection referred to as the eco traffic signal system. Li and Gao investigated optimal signal control strategies for fuel economy in a connected vehicle environment and showed that gasoline vehicles could achieve 10% emission reduction via such strategies [37]. Rakha et al. estimated potential emission impacts of vehicle-to-vehicle communication and signal coordination, and it turned out to be 8–23% emission savings depending on the vehicles’ traveling attributes [19,40]. 

The potential to reduce fuel consumption and GHG emission at the intersection is very high, as vehicles traveling near intersections at lower speeds tend to consume more fuel [41]. Yelchuru and Waller adopted micro-simulation models to estimate vehicle emissions under connected eco-traffic signal timing and the associated optimal signal timing plans [42]. According to the study, under a fully connected protocol, 2–6% emission reduction can be achieved in an average passenger vehicle. Zimmerman et al. compared traffic patterns before and after a user information system was introduced at different signalized intersections in Phoenix, Arizona [43]. The empirical data reported that the delay was reduced by 6.2%, resulting in a 1.8% emission reduction using vehicle speed profile and energy consumption correlation. As mentioned, signalized intersections in urban areas have the huge potential to reduce GHG emissions at the network level. AVs are equipped with different sophisticated sensors for communication with roadway surroundings that can guide the drivers/vehicles to adjust the driving patterns, minimize stops and speed variance. All these factors will reduce fuel consumption and hence vehicular emissions. 

### 3.4. Collision Avoidance

Human error accounts for more than 90% of accidents [44,45]. Collision avoidance systems in AVs are designed to provide necessary information ahead of time to the vehicle by means of well-designed vehicle mount sensors to avoid collisions. The sensors track nearby vehicles and objects to warn the system of preemptive maneuvers. In addition to the obvious individual advantages of accident avoidance, the system provides collective fuel-saving and environmental benefits by eliminating the chance of traffic congestion that might have arisen at a vehicle crash scene. According to Schrank et al., nationwide, 1.9% of GHG emission by the light duty vehicle (LDV) fleet was produced, due to the traffic congestion created at the accident spot [46]. Najm et al. integrated forward collision warning and adaptive cruise control functions to develop the ACAS for LDV applications [47]. The development of ACAS was based on an operational field test of 10 vehicle fleets driven by 66 drivers among diverse age and gender groups. The ACAS system has the potential to prevent about 10% of all rear-end crashes, which is expected to bring some indirect emission benefits. The collision avoidance attribute of both partial and full automation will reduce the GHG emissions, by preventing and minimizing jams and traffic congestion causing traffic accidents. 

### 3.5. Platooning

The vehicle platooning concept refers to the practice of multiple vehicles trailing closely enough to minimize aerodynamic drag to save energy and reduce vehicle emissions. Vehicle platooning can be safely and successfully implemented by leveraging automation and connectivity technologies. This strategy is particularly attractive considering that a significant portion of fuel consumption is attributed to confronting aerodynamic resistance while driving. Kasseris estimated that aerodynamic drag accounted for 50–75% of the tractive energy requirements for driving on a highway [48]. The shape of the vehicles in the convoy, distance headway, and order of the vehicles are the variables responsible for drag reduction in platooning. Since platooning advantage is more applicable to the vehicles in the middle of the pack, average fuel saving increases with the number of vehicles in the platoon. For two sedan cars running 1 m apart, the average reduction in drag has been estimated to be 10% [49]. Drag reductions ranging from 20% to 60% have been reported for platoons consisting of mixed vehicle types [50,51]. For a 3-truck platoon of freight trucks, Tsugawa has reported a 10% reduction in energy consumption at 80 km/h, with a 20 m gap between trucks; the reduction could reach up to 15% at 5 m gap [52]. The assumption that 50% tractive energy is used to overcome drag resistance could be combined to the advantage of vehicle platooning, which may yield an overwhelming 22.5–27.5% emission reduction. Zabat et al. also examined the potential of emission reduction in vehicle platooning through experiments done in a series of wind tunnels, along with numeric simulations using a passenger van [53]. They found that the average emission reduction per vehicle ranges from 10% to 30%, depending on the vehicles’ space in the platoon, number of vehicles, and other variables. Another study confirmed that when 15 vehicles are driving 6–8 m apart, they may achieve optimum fuel saving in the platoon, however, such a gap is extremely unsafe for conventional human-driven cars, but entirely within the capacities of autonomous vehicles [54]. It may be argued from the present literature that AVs vehicle platooning will lead to lower GHG transport emissions, primarily due to drag reduction and lower speed fluctuations. 

### 3.6. Vehicle Right-Sizing

Automation technologies have the potential to scale down the size of automobiles without compromising safety [22]. A significant improvement in fuel efficiency could be achieved by vehicle downsizing. The LDVs are designed to run on US roads with the least capacity of holding four passengers [22,55]. However, the average occupancy of these LDVs is only 1.67 in 2009 [56]. Once individual trip requirements are fulfilled, vehicle right-sizing can significantly reduce the average energy intensity. The vehicle size appropriation works best when it is coupled with car-sharing or carpooling. A fleet of shared AVs could easily supply the right-sized vehicle to meet passenger demand and discourage over-designed cars from being under-used [57]. MacKenzie et al. tested multiple conflicting influences on vehicle weight in terms of technological changes and functional improvement [58]. They indicated that progress in energy efficiency technology had been counterbalanced by increasing vehicle size and vehicle content. In particular, their study revealed that, for an average 2011 model car in the U.S., the safety-related features accounted for a total of 7.7% of the car’s weight, and dislodging them could result in a 5.5% reduction in emission. In general, a reduction of 20% in vehicular weight is attributed to a 20% increase in fuel efficiency [59]. The engine power required and amount of fuel consumed during a trip are proportional to the size of a vehicle. With AVs technologies in practice, manufacturers can scale down the vehicle sizes, leading to substantial energy and GHG emission benefits. 

### 3.7. Congestion Mitigation and Efficient Routing

As intermittent traffic experiences frequent stop-and-go and idling conditions, a car driving through heavy traffic will use more fuel, thus emitting more GHG than uncongested traffic. AVs will have the ability to coordinate with other vehicles and infrastructures (V2V and V2I) at the intersection, to improve the traffic flow and reduce the crash frequency that will result in less energy use and less GHG emission [22]. Bigazzi and Clifton’s study indicated that internal combustion engines (ICEs) fail to maintain fuel efficiency in slow-moving traffic at a speed of 30 miles per hour or lower [60]. In contrast, Gas electric hybrid vehicles are less sensitive to speed variations and retain fuel efficiency roughly at 20 mph. Though vehicles with different powertrain respond differently to congestion, an AV essentially powered by electricity has a higher potential of reducing GHS.

V2I technology available in AVs could also reroute cars within the road network in case of an unexpected influx of traffic into the grid network generated from a sports/entertainment event [61]. A fully developed city’s infrastructure is capable of receiving data from vehicles, anticipating traffic flows, and route vehicles with preference and faster routes given to emergency responders and school buses most efficiently [62]. Smart vehicle communication characteristics of AVs can give early warnings of traffic incidents and unanticipated traffic ahead. This will allow the vehicles to take optimal routes and smoothly flow through the network, and hence lower GHG emissions are released into the atmosphere. 

### 3.8. Carpooling

The occupancy rate is a key factor for GHG emissions associated with existing car travel. Fewer passengers per vehicle will result in more vehicles running on the road than required, and this will result in emissions increasing by several folds. For instance, only 11% of Americans carpool to work, and a staggering average of 113.6 million people make solo trips to and from work daily [63]. AVs have the potential to emerge as a new paradigm of business model to leverage the benefit of ridesharing, which would bring about a modal shift from individually owned vehicles to shared mobility services. Such changes are expected to reduce transportation GHGs significantly. AVs will also provide the option of carpooling and ridesharing that can lower GHGs emissions by reducing the auto-ownership, and travel through other less convenient transport modes. 

### 3.9. Traffic Law Adherence

Iglinksi and Babiak believe that autonomous vehicles will more strictly adhere to traffic laws as compared to the human driver, due to their integrated onboard programming logic [64]. AVs will be more likely to travel at posted speed limits designed to cater to optimal fuel efficiency, reducing GHGs considerably. Similarly, AVs will also strictly comply with traffic signals and thus reducing the nuisance and congestion created by human traffic. GHG reduction at different levels of vehicle automation reported in the literature are listed in Table 1. 

## 4. Causes of Increase in GHG Emissions

This section reviews some of the predominant factors that may increase GHG emissions due to vehicle automation. The impact of two-vehicle automation strategies, i.e., partial automation and full automation, will be discussed. 

### 4.1. Easier Travel

Easier travel involves reaching destinations more quickly due to capacity increases and fewer crashes, and lower travel costs. Travel may be faster and more reliable if crashes and congestion are reduced, and travel demand may increase. Capacity would effectively increase by less congestion and fewer crash delays, which could also trigger increased travel. Using activity-based travel model-generated scenarios, Childress et al. analyzed possible changes in travel patterns in the Puget Sound region [68]. These evaluated scenarios were comprised of a 30% increase in roadway capacity, resulting in a 3.6% increase in emissions, and a 35% reduction for the highest-income households in the perceived value of travel time cost. In a different scenario, assuming everyone owned an automated vehicle (no shared one), which resulted in a 30% increase in roadway capacity and 50% less parking costs, along with a 19.6% increase in emissions. People may be more likely to drive in automated vehicles under congested conditions. Easier travel means that more and more people will be attracted to use AVs, especially during traffic congestion situations. Greater demand and increase in road capacity will ultimately lead to increased vehicular emissions. 

### 4.2. Faster Travel

CAVs will be able to navigate and respond more quickly than human drivers with the state-of-the-art communication technology available onboard; it follows that AVs will be able to ride more safely at higher speeds than human drivers. AVs are expected to leverage V2V and V2I networks that communicate charted courses seamlessly to raise the speed limits on freeways [62]. To ensure a safe driving environment that accounts for operator reaction time, vehicle design, and road limitations, speed limits were initially imposed in the US, later changed at the federal level to minimize fuel consumption [32]. Therefore, an increase in fuel consumption is expected for increasing speed limits across the country due to AVs [22]. Considering driver’s value of time analysis, Wadud et al. analyzed the possible repercussions of increased highway travel speeds due to automation technologies [22]. A typical car’s speed-fuel consumption relationship was used to conclude that GHG emission of the highway could increase by 20–40% [72]. According to Brown et al., the increase in highway fuel use could be as high as 40% or more as a result of faster travel [73]. Brown et al. focused on travelers’ time budgets based on Schafer et al.’s observation that different societies display the same willingness to travel [35,74]. They hypothesized that if people could travel faster, they might prefer to live further away from their regular destinations, only to promote urban sprawl. Ultimately, this might trigger a possible increase in emissions by 50%. The onboard vehicle communication and sensing technologies of AVs will require a higher posted speed limit at the network level. It is established that faster travel is accompanied by greater fuel consumption, and hence the rate of GHG emissions. 

### 4.3. Increased Travel by Underserved Populations

Although access to mobility services to the disabled and people at dotage rendered by the AVs seems beneficial for society, it is likely to increase overall VMT. Due to the lack of adequate data on why some population groups travel less than others, it is difficult to forecast future travel patterns of those who are currently underserved. MacKenzie et al. observed from the 2014 National Household Travel Survey data that VMT for adults over 62 years old is much lower than the 42 years old group [58]. Fully automated vehicles could fulfill this travel demand. They estimated that increased travel could raise emissions by 2–10%. Harper et al. assumed that non-drivers would travel as much as drivers in each age group aged between 19–64; drivers with medical conditions are also expected to have similar travel patterns as drivers without medical conditions within each age group [70]. Dividing the sample population into three distinct groups of non-drivers 19 and older, elderly drivers without a medical condition, and drivers 19 and older with a medical condition, it was estimated that the underserved could increase emissions up to 12% by using fully automated vehicles. Examining data from the 2009 NHTS and the 2003 Bureau of Transportation Statistics publication “Freedom to Travel,” Brown et al. estimated a 40% increase in GHG emission, If all age segments traveled close to the top decile in each segment [35]. The fact that AVs can be used by non-drivers, people without driving licenses or people with special needs will increase the road user population and hence the daily number of vehicle trips. However, although it may have several positive prospects, GHGs are expected to increase. 

### 4.4. Mode Shift

The theory of travel behavior implies that the preference to use one mode over another is influenced by several variables, including, but not limited to, socio-economic status, age, gas price, urban form, and transportation options availability. Metropolitan Area Planning Council (MAPC) conducted a study in the Boston area, in which researchers found that those who use transit passes daily, or weekly, would replace transportation network companies for transit frequently. Frequent transit users are more likely to be willing to sacrifice the service in favor of a ride-sharing opportunity, even at a large difference in cost or forfeiting the money they already paid to avail the service [75]. A ride in a driver-less, fully autonomous vehicle will likely be cheaper [76,77]. New mobility services, and eventually autonomous vehicles, on the contrary, could increase ridership by solving the first-mile/last-mile problem and serving as a complement to mass transportation, thereby increasing GHG emissions. Shifting a staggering 56.5 billion miles (according to the National Transit Database for 2013) to vehicle-miles constitutes an increase in emissions of 2.0%. If it is assumed to be in city travel only, it accounts for an increase of 3.7% in city emission. Considering the change from air transport, an estimated 79.8 billion passenger miles traveled over domestic flights of less than 500 miles. Shifting all of these passenger-mile to non-shared vehicle-mile AVs in a possible scenario reflects a rise of 2.9% in emissions. However, this condition is projected to increase emissions only on highways. With AVs in operation at relatively lower journey costs than other transport modes, more and more people will be inclined to use AVs, which will also lead to high GHG emissions. 

### 4.5. Increased Empty Miles Traveled

AVs have not been extensively studied for potential changes in vehicle travel without a passenger. A vehicle owner could send his driverless AV to pick up family members or send nearby locations beforehand to minimize wait time. An agent-based model of self-driving vehicles moving in a square grid representing an imperial city was used by Fagnant and Kockelman to investigate the travel patterns of users of a shared fleet of self-driving vehicles [71]. With some predefined available data from 2009 NHTS, they examined scenarios with varying trip generation rates, level of network congestion, neighborhood size and vehicle relocation strategies. Finally, the study concluded that almost 11 conventional vehicles could be replaced by a self-driving vehicle with an increase of 5–11% in emission for vehicle repositioning. Vehicle idling while waiting for the passengers’ pick up from their destinations is the main source of increased vehicle miles traveled and resulting emissions. 

### 4.6. Land Use Change

Since individuals are liberated from the pressure of being behind the wheel and can use the time for work or recreation instead, there is a likelihood that they can accept longer commutes. For example, Cervero and Murakami observed data from 370 urbanized areas in the U.S. They deployed structural equation modeling to determine the relationship of population density with VMT per capita and found that an increase in population density leads to a decrease in per capita VMT [78]. When it comes to urban form, they pointed out a vital issue: traditionally, societies have been more reluctant to relocate residential roads or emphasize keeping the roads in the first place when built [79]. These findings indicate that if the introduction of AVs increases the pressure of growth in suburban areas, an increase in GHG emissions could result as people are concentrated in areas that facilitate more auto travel. Access of AVs to remote and sub-urban areas will encourage the public to opt for longer commutes and frequent travel, which will ultimately cause increased vehicular emissions at the network level. 

## 5. Change in GHG Emissions at Different AV Penetration Levels

This section investigates changes in emissions at different AV penetration levels using integrated traffic microsimulation and emission models. With better operating efficiency and improved powertrain technology, AVs are expected to yield overall emission benefits. Stogios et al. designed a study to evaluate the potential impacts that AVs could offer under varying scenarios [80]. Under interrupted and uninterrupted traffic flow conditions, high and low traffic conditions were evaluated. This study integrated the use of VISSIM microscopic software with the MOVES emission model to assess vehicular emissions. Eight inbuilt car-following and two lane-changing parameters present within the VISSIM model are investigated, representing AV driving behavior. The high traffic volume is reflected by an increase of 50% increase of the demand, while low traffic volume is produced by reducing the demand by 50%. A set of simulations is completed in the VISSIM model with 10%, 30%, 50%, 70%, and 90% of AVs penetration rate to investigate the changes in emission from the base condition. The study revealed that headway time has the highest impact on emissions and average delay than other parameters. Maximum headway time representing a cautious driving behavior resulted in a 31% increase in overall emissions, while a shorter headway time resembling aggressive driving behavior reduces the emission by 10%. The growing penetration of AVs into the network within high-traffic conditions results in minor incremental changes in emission factors and the number of stops per vehicle. In contrast, aggressive AVs reduce the average number of stops and emissions with increased market penetration. The AV penetration rate results, however, are not as evident under low traffic conditions. That is to conclude from the study that AVs will offer the maximum benefits under congested traffic conditions. 

Olia et al. deployed the PARAMICS microsimulation framework integrated with CMEM emission model to measure the vehicle emission at different market penetration of connected autonomous vehicles [81]. The CMEM model is capable of continuously estimating gas emissions and fuel consumption at the microscopic level. The emission and fuel consumption in the CMEM model vary based on vehicle type, age, fuel system, and emission control technology. The vehicles in this model were divided into three categories, unfamiliar non-connected, familiar non-connected and CVs to produce emission factors for CO_2_, CO, NOx and HC. The results showed that with a gradual increase of CVs market penetration, the emission factors decreased. The maximum emission benefit could be realized at 50% CV penetration, where the GHG emission is reduced by 30% from the base condition.

**Figure 2:**
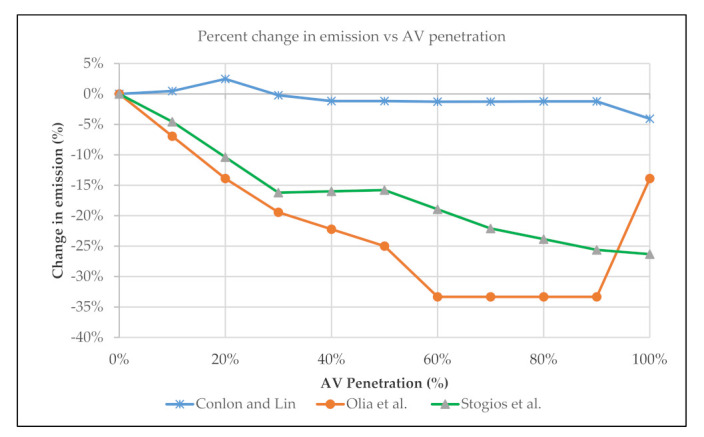
Emission changes by AV penetration [80,81,82].

Another study by Conlon and Lin attempted to quantify the changes in CO_2_ emission as the AVs are gradually penetrated into a congested urban road network [82]. SUMO traffic microsimulation and Newton-based greenhouse gas model (NGM) emission model were integrated to estimate the emission for different AV penetration, ranging from 0% to 100% into the network with an interval of 10%. At an AV penetration rate lower than 30%, the total CO_2_ emission had increased from the baseline of 0% AVs. The increase of total emission is explained by the difficulty in the interaction between human-driven vehicles (HDVs) and AVs. As the AVs penetration rate gradually increased, the study network started to realize the benefit of AVs in traffic operation, travel speed, and emission reduction. However, the emission reduction remained plateaued between a wide range of 40% to 90% AV penetration. Finally, at full AV penetration with no heterogeneity, the network was found to yield a maximum reduction of CO_2_ emission of 4.08% from the base condition. The changes in emission at different AV penetration levels from different studies could be compared for better understanding (Figure 2). Existing literature in this regard suggests that noticeable emission benefits of AVs at the network level can be achieved at penetration rates ranging between 30% and 50%.

## 6. Energy Consumption and GHG Emission

In recent years, the transportation sector has become the top GHG emitter surpassing electricity generation in the U.S. It accounted for approximately 28.5% of total atmospheric emissions in the country and continued to be the rapidly growing emissions source of any energy-related sector [83,84]. The global share of GHG from transportation is estimated to be around 24% of all emissions [85]. Passenger cars are accountable for 75% and 60% of transportation emissions worldwide and in the U.S., respectively [84,85]. The emergence of AVs can bring numerous energy and emission benefits, due to homogeneous traffic flows, lower highway congestion, lighter and smart vehicles shaped to minimize air resistance, minimum vehicle idling, the need for less powerful engines, etc. This would further enhance fuel efficiency and reduce emissions.

Similarly, shorter time spent searching for nearby parking and reduced needs for construction, operation, and maintenance of parking infrastructures could also bring various environmental benefits. Furthermore, the prospects that AVs serving passengers’ demand for performing various activities will be larger than traditional vehicles cannot be excluded. Under such circumstances, larger vehicle sizes may somehow limit fuel efficiency gains. However, shared AVs may be programmed to continuously drive rather than looking for parking in the city’s downtown until the next call for a ride, thus generating more emissions. This issue may be partially mitigated by programming the AVs to drive themselves outside of the downtown of an urban area where parking is free or relatively cheaper. However, this extra travel will lead to more energy consumption, creating more traffic congestion and subsequently producing more vehicular emissions. 

In the literature, numerous studies have discussed the prospects of fuel energy saving through vehicle automation. For example, Wu et al. reported that the deployment of a fuel economy optimization system could offer the automated systems or human drivers with essential guidance about optimal deceleration/acceleration profiles, taking into account vehicle current speed and acceleration, as well as other information such as headway spacing, signs, and traffic lights [86]. The authors conducted a driving simulator experiment in an urban setting through a network of signalized intersections and noted a nearly 31% reduction in fuel consumption for drivers using the system. Likewise, Khondaker and Kattan reported that a variable speed limit control algorithm resulted in approximately 16% fuel savings compared to an uncontrolled scenario [87]. The proposed control system integrated real-time intelligence about individual driver behavior (like the level of compliance with the established speed limits, acceleration/deceleration) in the situation of 100% connected vehicles (CVs) environment. However, fuel savings were only marginal at a penetration rate of CVs below 50%. In their study, Li et al. demonstrated that under automated car-following scenarios, the application of a pulse-and-gliding (PnG) controller could offer up to 20% savings in fuel compared to a conventional linear-quadratic (LQ)-based controller [88]. Other field tests and simulation studies have also shown that various types of adaptive cruise controller (ACC) and cooperative adaptive cruise controller (CACC) vehicle control algorithms could significantly reduce fuel energy consumption [89,90,91,92]. 

Zohdy and Rakha designed a controller equipped with CACC that can guide the optimum course of vehicles in the context of the urban road intersections network [93]. The study compared the fuel consumption for their system with various intersection geometries, and noted that on average, 11%, 45%, and 33% fuel saving were obtained compared to conventional intersection control approaches of a roundabout all-way-stop and traffic signal, respectively. In their studies, Kamalanathsharma, and Rakha; Asadi and Vahidi, and Ala et al. reported that the CACC that uses vehicles to infrastructure (V2I) communication to optimize vehicle trajectories in the vicinity could lead to a reduction in a fuel energy saving of about 47%, 30%, and 19%, respectively [94,95,96]. A recent study conducted by Manzie et al. also reported that a road-vehicle environment where vehicles can exchange traffic flow information via inter-vehicle communication and sensors could achieve about 15–25% savings in fuel consumptions [97]. They further stated that this number could reach as high as 33%, depending on the amount and quality of traffic information that they can process and exchange.

Similarly, in another study, Wang et al. observed that a higher penetration rate of intelligent vehicles equipped with a longitudinal vehicle controller was associated with lower NO_x_ emissions in a congested platoon [98]. Bose and Ioannou reported that a fleet containing only 10% ACC-equipped vehicles could lower NO_x_ emissions by 1.5% CO and CO_2_ emissions by up to 60% [99]. Choi and Bae examined the CO_2_ emissions profiles for manual and CVs under lane changing operations [100]. The study found that CVs can lead to 7.1% less CO_2_ emission, while lane change can maneuver faster to a slower lane. Likewise, lane change operations for CVs from a slower to a faster lane were associated with around 11.8% CO_2_ emissions benefits. Fagnant and Kockelman conducted a larger-scale agent-based study. They replicated a mid-sized city scenario where nearly 3.5% of the total trips on a given day are undertaken by shared AVs [71].

These researchers observed that autonomous vehicles could have a significant positive effect on reducing various pollutants (i.e., SO_2_, CO, NOx, volatile organic compounds (VOC), PM10, and GHG). VOCs and CO emissions were reduced the most, mainly due to the lower frequency of the vehicle’s cold start. Effects on the particulate matter with a diameter less than 10 mm (PM10) and GHG were comparatively insignificant due to the need for additional trips that shared vehicles have to make to pick up and drop off passengers from different locations. However, it is worth mentioning that this simulation study was limited by the assumptions that automated vehicles in the fleet are not essentially powered by electricity, hybrid-electric, or running on alternative fuel and passengers would not make trips more frequently. The long-term effect of automated vehicle-related emission reduction could realize a very optimistic level, as indicated in a study by Greenblatt and Saxena that estimated the emission of shared electric autonomous taxis. The study found that the GHG reduction per vehicle per mile in 2030 could be 87–94% less than the emissions of gasoline-based internal combustion vehicles in 2014 and 63–82% less compared to hybrid-electric vehicle emissions in 2030 [101]. 

Brown et al. also predicted considerable energy-saving up to 91% per automated vehicle in 2030 in a framework that accounted for the highest impact of energy-saving factors (e.g., efficient travel, electrification and optimized vehicle weight) and increased energy use (e.g., increased travel distance by dependent traveler) [35]. However, the factors and to what extent they will offer emission benefit in the future remains an open question. As a result, the trade-off between energy savings and increased energy use from automated vehicles might fluctuate substantially. 

Few studies have also argued that the benefit in emission reduction by AVs could be fully offset by increased travel, due to lower costs involved in travelling. A study by Taiebat et al. used microeconomic modeling and applied econometric techniques to analyze the travel and energy impacts of CAVs with respect to the price of fuel and travel time [102]. While increased fuel economy in CAVs reduces the amount of energy required per mile traveled, it also decreases the cost of travel, encouraging additional travel and leading to an energy “rebound effect.” The elasticities of VMT demand with respect to fuel and time costs were estimated using the developed microeconomic model under income and time constraints. The forecasted travel demand for a typical household was estimated to increase by 2–47%. Numerous plausible scenarios involving changes in fuel economy and time costs resulted in an overall increase in energy consumption. In higher-income quantiles, backfire is more likely as the reduction in time cost is less appreciated in this class, only to offset the energy savings from CAVs. On average, a 38% reduction in time costs completely offsets a 20% increase in fuel economy provided by CAVs. Numerous researchers have also pointed out that the higher penetration of automated vehicles may actually increase the vehicle fleet number and contribute to the rise of GHGs in the environment [103]. The burgeoning number of automated on-demand mobility or ride-hailing services may lead to an enlargement of the number of vehicles in the fleet, increased VMTs and road congestion, and thereby increased fuel consumption and GHG emissions. 

**Figure 3:**
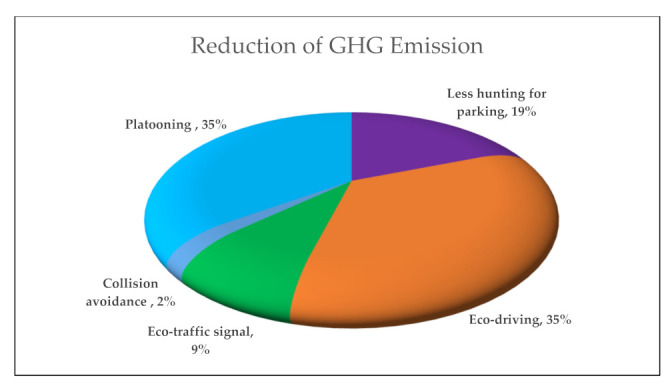
Average contribution of the causes on GHG emission reduction.

**Figure 4:**
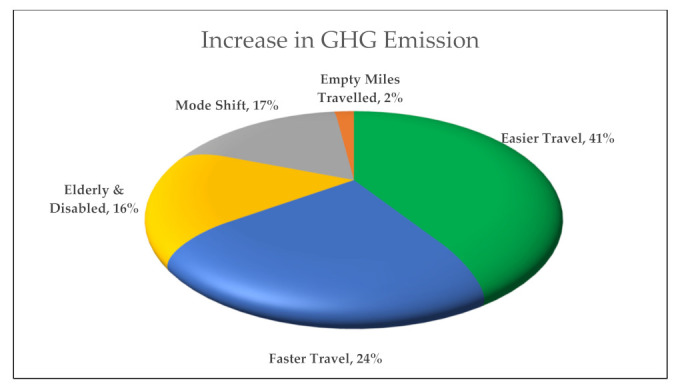
Average contribution of the causes on GHG emission increase.

**Figure 5:**
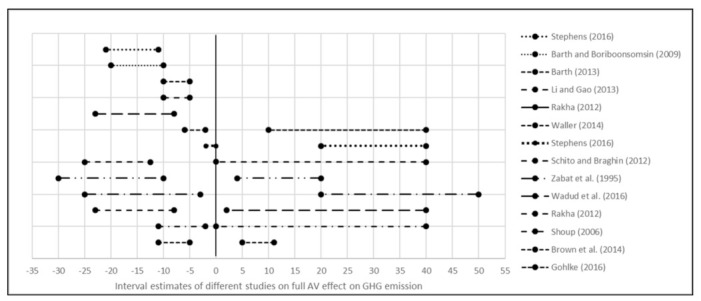
Interval estimates of different studies on full AV effects on GHG emission.

Synthesizing the result of all the previous studies, some charts could be developed to better understand and visualize the results of the level of GHG decrease or increase. The first graph (Figure 3) shows the factors that will increase emissions, while others are for the factors that will reduce the emission (Figure 4). In the last chart, Figure 5 demonstrates the result ranges for all research studies. 

### 6.1. Causal Loop Diagram (CLD) of the AV’s Effect on GHG Emission

In transport studies, system dynamics have been applied, as the feedback and connections provided by these models are useful for defining interactions of variables within the transport system. Shepherd provided a review of the different system dynamics modeling approaches used in transport systems [104]. In his study, he mentioned that the causal loop diagram (CLD) is the primary technique used to analyze the qualitative relationships between various aspects of the system within system dynamics modeling. CLD is a helpful tool to explore possible sources of dissent to strategies, synergies, and repercussions within the system. Such prospects will then help identify potential problem statements that can be addressed by quantitative modeling. A CLD illustrates how important variables of the system interrelate with each other by using text, arrows and symbols. Arrow running from the “cause” to the “effect” with a polarity represents the interaction between two variables, known as a causal connection. A positive polarity indicates that deviations in the “causal” variable would result in deviations in the “effect” variable in the same direction, assuming all other influences remain constant in the system. Similarly, a negative arrow shows that changes in one variable cause the other to change in the opposite direction, given that all other conditions are fixed. 

The feedback loops created by the causal relationship are termed as balancing (B) or reinforcing (R) based on the polarity sign, which represents positive or negative feedbacks, respectively within the system [105].

**Figure 6:**
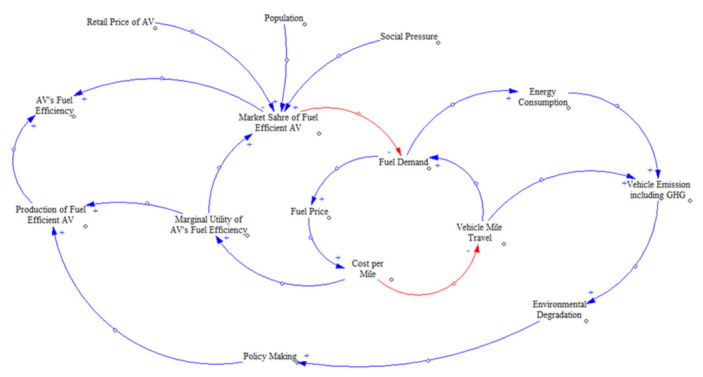
Causal Loop Diagram of the influence of fuel-efficient AVs on GHG emissions (inspired by [106,107]).

A CLD is developed based on the literature to depict the interactions of different root causes and variables with the GHG emissions from AVs (Figure 6). The CLD starts with the gradual penetration or increased market share of AVs within the transportation system. This system dynamic model assumes that both the non-AVs and AVs use fossil fuel for power generation. Since the AVs are fuel-efficient, there is a substantial chance that the demand for AVs increases, with all its benefits in terms of traffic safety, operation, and management. However, since the AVs are expected to offer several benefits to the transport system, the introductory retail price of it might be some fold higher than the conventional non-AVs. A higher retail price of AV will impart a negative effect on AV’s market share.

Nevertheless, the increase in population and social pressure to purchase AVs will positively affect the AV’s penetration rate to the market. In this context, it is predicted that the number of cars in the city will increase as the population increases, causing road congestion as well. Congestion reduces the efficiency of automobile engines, contributing to increased fuel consumption and leading to higher rates of pollution [107]. An increased market share of fuel-efficient AVs will reduce the fuel demand as a whole. The reduced fuel demand initiates a balancing loop; a shortfall of demand will push the fuel price to increase and increase travel cost per mile, only to be balanced by less miles traveled. The price of gasoline is a wiggle that can play either in favor or against AVs. As observed today, gasoline prices have not prevented the ownership and use of fossil fuel vehicles (FFV) in general, but if prices go up, FFV use could fall as people move to more affordable choices, given the limited nature of petrol resources. However, an increase in the cost/miles travel will observe fuel-efficient AVs’ marginal utility as people will enjoy the added benefit by buying an additional AV unit. 

A reinforcing loop will also generate fuel demand. In the event of increased demand, energy consumption will also escalate, giving rise to vehicle emission or GHG emission. Implementing pollution reduction policies that cause environmental degradation should be balanced in this loop, though there is a delay in this cycle that prevents it from performing as planned. The mounting pressure on policy regulation to control the environmental degradation will possibly deter the growing AV production. More capital is expected to be invested within the automobile industry to make the AVs more fuel-efficient.

### 6.2. AVs Potential Impact on Reducing GHG Emission during a Global Pandemic

On 30 January 2020, the World Health Organization (WHO) announced the respiratory coronavirus disease outbreak 2019 (COVID-19) and subsequently, on 13 March, declared a global pandemic. While government policies in most countries reduced mobility, travel also declined in response to the number of local cases in the respective country. This shows how people adapted their travel behavior depending on the level of information available on the outbreak. Not only did people restrict their travel, but destinations were often avoided that had more infected cases. The automotive and transport industries are closely observing how consumer behavior changes will impact AV technologies in key aspects of the economy and daily life, given that numerous changes have been imposed upon people’s daily lives due to the global COVID-19 pandemic. 

COVID-19 is overhauling the consumer’s perceptions towards public transit in ways that are likely to support AV technology in the longer run. As the pandemic has spread across the world, people have generally remained home, either by choice or by local directives. Hence, transit ridership has declined substantially, barring essential and emergency support workers. Major cities like New York, Washington, D.C., and San Francisco of the US have seen the ridership plummeted by a staggering 70–90% in August 2020 compared to the same time in the previous year [108]. While the decrease in ridership is attributed to home-based work, the closure of educational institutes, and local travel bans, consumers have become more interested in personal motor vehicle ownership than ever before. While the potential car customer might be putting new purchases on hold, McKinsey’s recent survey reported that “20 percent of people in the United States who do not possess a vehicle under their name, now considering buying one” [108]. This group mainly includes people who live in cities and rely on public transportation for mobility. While the customer demands for new and used cars may have temporarily postponed adopting AV systems in the consumer sector, the COVID-19 pandemic per se warranted the important role of AV in day-to-day business and, most importantly, to deal with the risks posed by COVID-19.

Over the past decade, the automotive industry has had to adapt to changing attitudes to mobility, with global car ownership predicted to peak in 2034 before beginning its decline. However, with many still reluctant to use public transport due to the risk of infection, the prospect of owning a car may seem more inviting in the context of the unprecedented COVID-19 pandemic. This change in attitudes towards mobility is already evident in the adoption of micro-mobility solutions, while some have predicted that autonomous vehicles, capable of driving with some to no human input, may see an acceleration in terms of development, deployment and public interest. With industrial activity forced to slow down, flight and car journeys decreasing, greenhouse gas emissions around the world have plummeted. Consumers will get used to these changes, which is likely to see an increase in the adoption of autonomous vehicles in the future. These new vehicles are meant to be fuel-efficient, affordable, clean and green and a natural feature in smart cities and interactive communities—and will forever change the future of mobility. One of the key barriers to autonomous vehicle rollout is public perception, with a 2018 survey by OpenText revealing that 52% of consumers would not buy a driverless car. However, the COVID-19 pandemic may have contributed to changing attitudes. When weighing up the risk of COVID-19 infection presented by public transport or shared mobility, it is possible that the public will look more favorably on driverless cars. The current pandemic has had a significant impact on transport demand and mode, with a shift away from shared mobility, and in particular public transport, because of worries over public health.

## 7. Conclusions

Net effects of vehicle automation on emissions across a variety of illustrative examples show that automation could theoretically reduce GHG emissions and energy usage plausibly by almost half—or double-fold—depending on the implications that would come to the fore [22]. It is believed that reductions in GHG emissions through AVs’ adoption will be negated to an unascertained extent, mainly due to increased car travel, facilitated by other factors such as lower perceived travel time and costs per km/trip, probable loss of public transport patronage, and possible increases in car ownership. Thus, it is quite possible that AVs could be more energy-efficient, thereby reducing the GHG by functional unit-basis as per-passenger-mile (ppm); however, the overall gain related to transportation GHG emissions could be swamped by a surge in increased vehicle miles traveled (VMT).

The effect of AV adoption on consumer travel patterns could be more pronounced from environmental aspects rather than technical attributes. While it is challenging to accurately estimate the behavioral fronts to AV adoption, a more tangible consideration of the relationship between different AV adoption models and anticipated travel behavior is vital for estimating AVs’ environmental impacts. It may be argued from the discussion presented herein that if AVs are deployed within less approbatory areas or if the road transportation sector is continued to be dominated by privately owned vehicles, it is likely that AVs may escalate the transport-related GHG emissions. Hence, adoption tendencies like vehicle ownership models are also expected to largely influence whether AVs will decrease or increase the overall VMT as well as the subsequent GHG emissions. Few studies have indicated that the positive emission changes may not be realized at lower AV penetration rate, where the maximum emission reduction might take place within the 60–80% AV penetration rate.

Impacts of autonomous vehicles on GHG emission are highly dependent on continuous technological development and evolution, market reaction, and regulatory actions, making it challenging to confidently predict the overall benefits expected to deliver by AVs to the transportation systems in terms of GHG emission. With long-term land-use adjustments, the role of policy, welfare and equity yet to be explored and the potential effects of AVs remain unknown; it is unlikely that we can anticipate long-term effects on GHG emission with certainty. Moreover, the overwhelming COVID-19 global pandemic has also posed challenges to some of the well-perceived mode choice models, which may force the policymaker to adopt suitable mobility alternatives that ensure public health and safety. Therefore, it is of paramount importance to develop appropriate methodologies, tools, and techniques to better understand the impact of GHG emissions for AV adoption at different levels by harnessing an appropriate system approach.

## Figures and Tables

**Table 1:** Reduction of GHG emission at different levels of vehicle automation.

Study	Level of Automation	Cause of Reduction in GHG	Results	Condition
Stephens (2016) [17]	Partial Automation	Driver profile and Traffic flow calming	0–10% 0–5%	During peak hours During non-peak hours
	Full Automation		10–21% 5–11%	During peak hours During non-peak hours
Barth and Boriboonsomsin (2009) [15]	Full Automation	Eco-driving	10–20% nearly 0%	Congested highway traffic. Free flow
Xia et al. (2013) [65]			5–10%	Under congested city traffic
Li and Gao (2013) [37]			10%	Under congested city traffic
Rakha (2012) [40]			8–23%	Under different speed, congestion level and design characteristics
Yelchuru (2014) [42]	Partial automation	Eco-traffic signal timing V2i/i2v communication	1.8–2%	City driving
	Full Automation		2–6%	City driving
Schrank et al. (2012) [46]	Partial Automation	Collision avoidance	0–0.95%	City driving
Stephens (2016) [17]	Full Automation		0–1.9%	
Stephens (2016) [17]	Partial Automation	Platooning	0–12.5%	During peak hours
Schito (2012) [50]	Full Automation		12.5–25%	During non-peak hours
			22.5–27.5%	During non-peak hours
Zabat et al. (1995) [53]			10% to 30%	During peak hours
			20–25%	During non-peak hours
Wadud et al. (2016) [22]			3% to 25%	During non-peak hours
Wadud et al. (2016) [22]	Full Automation	Vehicle/powertrain resizing	45%–	No condition mentioned
Burns et al. (2013) [66]			roughly 50%	
Shoup (2006) [34]	Full Automation	Less Hunting for Parking	2–11%	During city driving
Brown et al. (2014) [35]	Full Automation		5–11%	
Barth (2009) [15]	Partial Automation		2–5%	
Brown et al. (2014) [35]	Full Automation	Increase in Ridesharing	Roughly 12%	During city driving
Stephens (2016) [17]	Partial Automation	Faster travel	0–10%	During peak hours
	Full Automation		10–40%	During non-peak hours
Haan et al. (2007) [67]	Full Automation		20–40%	During non-peak hours
Brown et al. (2014) [35]	Full Automation		0–40%	During non-peak hours
	Partial Automation		0–10%	During non-peak hours
Stephens (2016) [17]	Partial Automation	Easier travel	4–13%	No condition mentioned
Stephens (2016) [17]	Full Automation		30–156%	Living farther
Childress et al. (2015) [68]	Full Automation		3.6–19.6%	Capacity will increase and value of travel time cost will reduce
Gucwa (2014) [69]	Partial Automation		4–8%	Living farther
Brown et al. (2014) [35]	Full Automation		50%	
MacKenzie et al. (2014) [58]	Partial Automation		4–13%	
Stephens (2016) [17]	Full Automation	Increased Travel by Underserved Populations	2–40%	Elderly and disabled would travel as much as drivers without medical conditions
MacKenzie et al. (2014) [58]	Partial Automation	Mode Shift from Walking, Transit and Regional Air	2–10%	No condition mentioned
Harper et al. (2016) [70]	Partial Automation		Up to 12%	
Brown et al. (2014) [35]	Full Automation		Up to 40%	
Fagnant and Kockelman (2014) [71]	Full Automation	Increased empty miles travelled	5% to 11%	On city driving

## References

[1] Vahidi A., Sciarretta A. (2018). Energy Saving Potentials of Connected and Automated Vehicles. Transp. Res. Part C Emerg. Technol..

[2] Facts E.F. (2015). US Transportation Sector Greenhouse Gas Emissions 1990−2013.

[3] National Academies of Sciences, Engineering, and Medicine (2016). Commercial Motor Vehicle Driver Fatigue, Long-Term Health, and Highway Safety: Research Needs.

[4] Metcalf G.E. (2009). Market-Based Policy Options to Control US Greenhouse Gas Emissions. J. Econ. Perspect..

[5] Andrés L., Padilla E. (2018). Driving Factors of GHG Emissions in the EU Transport Activity. Transp. Policy.

[6] Liu D., Yang D., Huang A. (2021). LEAP-Based Greenhouse Gases Emissions Peak and Low Carbon Pathways in China’s Tourist Industry. Int. J. Environ. Res. Public Health.

[7] Crayton T.J., Meier B.M. (2017). Autonomous Vehicles_ Developing a Public Health Research Agenda to Frame the Future of Transportation Policy. J. Transp. Health.

[8] Newman P., Kenworthy J. (1999). Sustainability and Cities: Overcoming Automobile Dependence.

[9] Mersky A.C., Samaras C. (2016). Fuel Economy Testing of Autonomous Vehicles. Transp. Res. Part C.

[10] Moriarty P., Wang S.J. (2017). Could Automated Vehicles Reduce Transport Energy?. Energy Procedia.

[11] Markit I. (2016). Autonomous Vehicle Sales Forecast to Reach 21 Mil. Globally in 2035, According to IHS Automotive.

[12] Rivera J., van der Meulen R. (2014). Gartner Says 4.9 Billion Connected ‘Things’ Will Be in Use in 2015.

[13] Alexander-Kearns M., Peterson M., Cassady A. The Impact of Vehicle Automation on Carbon Emissions. Center for American Progress. https://www.americanprogress.org/issues/green/reports/2016/11/18/292588/theimpact-of-vehicle-automation-on-carbon-emissions-where-uncertainty-lies.

[14] Litman T. (2016). Safer Than You Think!: Revising the Transit Safety Narrative.

[15] Barth M., Boriboonsomsin K. (2009). Energy and Emissions Impacts of a Freeway-Based Dynamic Eco-Driving System. Res. Part D Transp..

[16] Chen T., Kockelman K., Hanna J.P. (2016). Operations of a Shared, Autonomous, Electric Vehicle Fleet: Implications of Vehicle & Charging Infrastructure Decisions. Transp. Res. Part A Policy Pract..

[17] Stephens T., Gonder J., Chen Y., Lin Z., Liu C., Gohlke D. (2016). Estimated Bounds and Important Factors for Fuel Use and Consumer Costs of Connected and Automated Vehicles.

[18] Barkenbus J.N. (2009). Author’s Personal Copy Eco-Driving: An Overlooked Climate Change Initiative.

[19] Rakha H., Kamalanathsharma R.K. (2011). Eco-Driving at Signalized Intersections Using V2I Communication.

[20] Psaraki V., Pagoni I., Schafer A. (2012). Techno-Economic Assessment of the Potential of Intelligent Transport Systems to Reduce CO2 Emissions. IET Intell. Transp. Syst..

[21] Pettigrew S., Fritschi L., Norman R. (2018). The Potential Implications of Autonomous Vehicles in and around the Workplace. Int. J. Environ. Res. Public Health.

[22] Wadud Z., MacKenzie D., Leiby P. (2016). Help or Hindrance? The Travel, Energy and Carbon Impacts of Highly Automated Vehicles. Transp. Res. Part A Policy Pract..

[23] Greenblatt J.B., Shaheen S. (2015). Automated Vehicles, On-Demand Mobility, and Environmental Impacts. Curr. Sustain. Renew. Energy Rep..

[24] Anderson J., Nidhi K., Stanley K., Sorensen P. (2014). Autonomous Vehicle Technology: A Guide for Policymakers.

[25] Tomás R.F., Fernandes P., Macedo E., Bandeira J.M., Coelho M.C. (2020). Assessing the Emission Impacts of Autonomous Vehicles on Metropolitan Freeways. Transp. Res. Procedia.

[26] Stasinopoulos P., Shiwakoti N., Beining M. (2021). Use-Stage Life Cycle Greenhouse Gas Emissions of the Transition to an Autonomous Vehicle Fleet: A System Dynamics Approach. J. Clean. Prod..

[27] Wang A., Stogios C., Gai Y., Vaughan J., Ozonder G., Lee S., Posen I.D., Miller E.J., Hatzopoulou M. (2018). Automated, Electric, or Both? Investigating the Effects of Transportation and Technology Scenarios on Metropolitan Greenhouse Gas Emissions. Sustain. Cities Soc..

[28] Le Hong Z., Zimmerman N. (2021). Air Quality and Greenhouse Gas Implications of Autonomous Vehicles in Vancouver, Canada. Transp. Res. Part D Transp. Environ..

[29] Liu F., Zhao F., Liu Z., Policy H.H.-E. (2019). Can Autonomous Vehicle Reduce Greenhouse Gas Emissions? A Country-Level Evaluation.

[30] de Souza Melaré A.V., González S.M., Faceli K., Casadei V. (2017). Technologies and Decision Support Systems to Aid Solid-Waste Management: A Systematic Review. Waste Manag..

[31] Kitchenham B., Charters S. (2007). Guidelines for Performing Systematic Literature Reviews in Software Engineering.

[32] Guccione L., Holland B. (2013). Car & (No) Driver.

[33] Wang W., Zhong H., Zeng Y., Liu Y., Chen J. (2021). A Carbon Emission Calculation Model for Roadside Parking. Int. J. Environ. Res. Public Health.

[34] Shoup D.C. (2006). Cruising for Parking. Transp. Policy.

[35] Brown A., Gonder J., Repac B. (2014). An Analysis of Possible Energy Impacts of Automated Vehicles.

[36] Torbert R., Herrschaft B. (2016). Driving Miss Hazy: Will Driverless Cars Decrease Fossil Fuel Consumption?.

[37] Li J.-M., Gao Z. (2013). Exploring the Impact of Speed Synchronization through Connected Vehicle Technology on Fleet-Level Fuel Economy. SAE Int. J. Passeng. Cars Electron. Electr. Syst..

[38] Rakha H.A., Ahn K., Park S. (2012). AERIS: Eco-Driving Application Development and Testing (No. FHWA). https://vtechworks.lib.vt.edu/handle/10919/55092.

[39] He Y., Rios J., Chowdhury M., Pisu P., Bhavsar P. (2012). Forward Power-Train Energy Management Modeling for Assessing Benefits of Integrating Predictive Traffic Data into Plug-in-Hybrid Electric Vehicles. Transp. Res. Part D Transp. Environ..

[40] Rakha H., Kamalanathsharma R., Ahn K. (2012). AERIS: Eco-Vehicle Speed Control at Signalized Intersections Using I2V Communication Final Report.

[41] Al-Turki M., Jamal A., Al-Ahmadi H.M., Al-Sughaiyer M.A., Zahid M. (2020). On the Potential Impacts of Smart Traffic Control for Delay, Fuel Energy Consumption, and Emissions: An NSGA-II-Based Optimization Case Study from Dhahran, Saudi Arabia. Sustainability.

[42] Yelchuru B., Waller T. Preliminary Eco-Traffic Signal Timing Modeling Results; 2014. https://www.itsbenefits.its.dot.gov/ITS/benecost.nsf/SummID/B2014-00912.

[43] Zimmerman C., Marks J., Jenq J., Cluett C., DeBlasio A. (2000). Phoenix Metropolitan Model Deployment Initiative Evaluation Report (Draft).

[44] NHTSA (2008). Fatality Analysis Reporting System.

[45] Zahid M., Chen Y., Khan S., Jamal A., Ijaz M., Ahmed T. (2020). Predicting Risky and Aggressive Driving Behavior among Taxi Drivers: Do Spatio-Temporal Attributes Matter?. Int. J. Environ. Res. Public Health.

[46] Schrank D. (2012). TTI’s 2012 Urban Mobility Report.

[47] Najm W., Stearns M., Howarth H., Koopmann J., Hitz J. (2006). Evaluation of an Automotive Rear-End Collision Avoidance System.

[48] Kasseris E.P. (2006). Comparative Analysis of Automotive Powertrain Choices for the Near to Mid-Term Future.

[49] Zhu H., Yang Z. (2011). Simulation of the Aerodynamic Interaction of Two Generic Sedans Moving Very Closely.

[50] Schito P. (2012). Numerical and Experimental Investigation on Vehicles in Platoon. SAE Int. J. Commer. Veh..

[51] Duan K., Mcdaniel C., Muller A., Yokota B., Kleissl J. (2007). Effects of Highway Slipstreaming on California Gas Consumption.

[52] Tsugawa S. (2013). An Overview on an Automated Truck Platoon within the Energy ITS Project. Ifac Proc. Vol..

[53] Zabat M., Stabile N., Farascaroli S. (1995). UC Berkeley Research Reports Title the Aerodynamic Performance of Platoons.

[54] Dávila A., Nombela M. (2011). Sartre-Safe Road Trains for the Environment Reducing Fuel Consumption through Lower Aerodynamic Drag Coefficient.

[55] Mccarthy J.F. (2017). Sustainability of Self-Driving Mobility: An Analysis of Carbon Emissions Between Autonomous Vehicles and Conventional Modes of Transportation.

[56] Davis S., Diegel S., Boundy R. (2012). Transportation Energy Data Book Edition 31.

[57] Organisation for Economic Co-Operation and Development (2015). Urban Mobility System Upgrade: How Shared Self-Driving Cars Could Change City Traffic.

[58] MacKenzie D., Zoepf S., Heywood J. (2014). Determinants of US Passenger Car Weight. Int. J. Veh. Des..

[59] Joost W.J. (2012). Reducing Vehicle Weight and Improving US Energy Efficiency Using Integrated Computational Materials Engineering. Jom.

[60] Bigazzi A.Y., Clifton K.J. (2015). Modeling the Effects of Congestion on Fuel Economy for Advanced Power Train Vehicles. Transp. Plan. Technol..

[61] Dey K.C., Rayamajhi A., Chowdhury M., Bhavsar P., Martin J. (2016). Vehicle-to-Vehicle (V2V) and Vehicle-to-Infrastructure (V2I) Communication in a Heterogeneous Wireless Network–Performance Evaluation. Transp. Res. Part C Emerg. Technol..

[62] Porter C., Brown A., DeFlorio J., McKenzie E., Tao W. (2013). Effects of Travel Reduction and Efficient Driving on Transportation: Energy Use and Greenhouse Gas Emissions.

[63] Shaheen S., Cohen A., Bayen A. (2018). The Benefits of Carpooling.

[64] Igliński H., Babiak M. (2017). Analysis of the Potential of Autonomous Vehicles in Reducing the Emissions of Greenhouse Gases in Road Transport. Procedia Eng..

[65] Xia H., Wu G., Boriboonsomsin K., Barth M.J. Development and Evaluation of an Enhanced Eco-Approach Traffic Signal Application for Connected Vehicles. Proceedings of the 16th International IEEE Conference on Intelligent Transportation Systems (ITSC 2013).

[66] Burns L. (2013). Sustainable Mobility: A Vision of Our Transport Future. Nature.

[67] De Haan P., Peters A., Scholz R.W. (2007). Reducing Energy Consumption in Road Transport through Hybrid Vehicles: Investigation of Rebound Effects, and Possible Effects of Tax Rebates. J. Clean. Prod..

[68] Childress S., Nichols B., Charlton B.C.-T. (2015). Using an Activity-Based Model to Explore the Potential Impacts of Automated Vehicles. Transp. Res. Rec..

[69] Gucwa M. Mobility and Energy Impacts of Automated Cars. Proceedings of the Automated Vehicles Symposium.

[70] Harper C.D., Hendrickson C.T., Mangones S., Samaras C. (2016). Estimating Potential Increases in Travel with Autonomous Vehicles for the Non-Driving, Elderly and People with Travel-Restrictive Medical Conditions.

[71] Fagnant D.J., Kockelman K.M. (2014). The Travel and Environmental Implications of Shared Autonomous Vehicles, Using Agent-Based Model Scenarios.

[72] Berry I.M. (2007). The Effects of Driving Style and Vehicle Performance on the Real-World Fuel Consumption of U.S. Light-Duty Vehicles.

[73] Thomas J., Hwang H.-L., West B., Huff S. (2013). Predicting Light-Duty Vehicle Fuel Economy as a Function of Highway Speed. Sae Int. J. Passeng. Cars Mech. Syst..

[74] Schäfer A., Heywood J., Jacoby H., Waitz I. (2009). Transportation in a Climate-Constrained World.

[75] Gehrke S., Felix A., Metropolitan Area Planning Council (2018). Fare Choices: A Survey of Ride-Hailing Passengers in Metro Boston. https://pdxscholar.library.pdx.edu/trec_seminar/152/.

[76] Whitney J. (2018). Autonomous Vehicles: How US Cities Are Preparing.

[77] Ullah I., Jamal A., Subhan F. (2019). Public Perception of Autonomous Car: A Case Study for Pakistan. Adv. Transp. Stud..

[78] Cervero R., Murakami J. (2010). Effects of Built Environments on Vehicle Miles Traveled: Evidence from 370 US Urbanized Areas. Environ. Plan. A.

[79] Barrington-Leigh C., Millard-Ball A. (2017). More Connected Urban Roads Reduce US GHG Emissions. Environ. Res. Lett..

[80] Stogios C., Kasraian D., Roorda M.J., Hatzopoulou M. (2019). Simulating Impacts of Automated Driving Behavior and Traffic Conditions on Vehicle Emissions. Transp. Res. Part D Transp. Environ..

[81] Olia A., Abdelgawad H., Abdulhai B., Razavi S.N. (2016). Assessing the Potential Impacts of Connected Vehicles: Mobility, Environmental, and Safety Perspectives. J. Intell. Transp. Syst..

[82] Conlon J., Lin J. (2019). Greenhouse Gas Emission Impact of Autonomous Vehicle Introduction in an Urban Network. Transp. Res. Rec..

[83] (2018). Bloomberg New Energy Finance.

[84] EPA (2013). US Transportation Sector Greenhouse Gas Emissions: 1990–2011.

[85] (2018). Global Energy & CO_2_ Status Report 2017.

[86] Wu C., Zhao G., Ou B. (2011). A Fuel Economy Optimization System with Applications in Vehicles with Human Drivers and Autonomous Vehicles. Transp. Res. Part D Transp. Environ..

[87] Khondaker B., Kattan L. (2015). Variable Speed Limit: A Microscopic Analysis in a Connected Vehicle Environment. Transp. Res. Part C Emerg. Technol..

[88] Li S.E., Peng H., Li K., Wang J. (2012). Minimum Fuel Control Strategy in Automated Car-Following Scenarios. IEEE Trans. Veh. Technol..

[89] Kamal M.A.S., Taguchi S., Yoshimura T. (2016). Efficient Driving on Multilane Roads under a Connected Vehicle Environment. Ieee Trans. Intell. Transp. Syst..

[90] Luo L., Liu H., Li P., Wang H. (2010). Model Predictive Control for Adaptive Cruise Control with Multi-Objectives: Comfort, Fuel-Economy, Safety and Car-Following. J. Zhejiang Univ. Sci. A.

[91] Rios-Torres J., Malikopoulos A.A. (2016). Automated and Cooperative Vehicle Merging at Highway On-Ramps. IEEE Trans. Intell. Transp. Syst..

[92] Wang M., Daamen W., Hoogendoorn S., Van Arem B. (2014). Potential Impacts of Ecological Adaptive Cruise Control Systems on Traffic and Environment. Iet Intell. Transp. Syst..

[93] Zohdy I.H., Rakha H.A. (2016). Intersection Management via Vehicle Connectivity: The Intersection Cooperative Adaptive Cruise Control System Concept. J. Intell. Transp. Syst..

[94] Kamalanathsharma R.K., Rakha H.A. (2016). Leveraging Connected Vehicle Technology and Telematics to Enhance Vehicle Fuel Efficiency in the Vicinity of Signalized Intersections. J. Intell. Transp. Syst..

[95] Asadi B., Vahidi A. (2010). Predictive Cruise Control: Utilizing Upcoming Traffic Signal Information for Improving Fuel Economy and Reducing Trip Time. Ieee Trans. Control Syst. Technol..

[96] Ala M.V., Yang H., Rakha H. (2016). Modeling Evaluation of Eco–Cooperative Adaptive Cruise Control in Vicinity of Signalized Intersections. Transp. Res. Rec..

[97] Manzie C., Watson H., Halgamuge S. (2007). Fuel Economy Improvements for Urban Driving: Hybrid vs. Intelligent Vehicles. Transp. Res. Part C Emerg. Technol..

[98] Wang Z., Chen X., Ouyang Y., Li M. (2015). Emission Mitigation via Longitudinal Control of Intelligent Vehicles in a Congested Platoon. Comput. Aided Civ. Infrastruct. Eng..

[99] Bose A., Ioannou P. (2001). Evaluation of the Environmental Effects of Intelligent Cruise Control Vehicles. Transp. Res. Rec..

[100] Choi J.-E., Bae S.-H. (2013). Development of a Methodology to Demonstrate the Environmental Impact of Connected Vehicles under Lane-Changing Conditions. Simulation.

[101] Greenblatt J.B., Saxena S. (2015). Autonomous Taxis Could Greatly Reduce Greenhouse-Gas Emissions of US Light-Duty Vehicles. Nat. Clim. Chang..

[102] Taiebat M., Stolper S., Xu M. (2019). Forecasting the Impact of Connected and Automated Vehicles on Energy Use: A Microeconomic Study of Induced Travel and Energy Rebound. Appl. Energy.

[103] Kopelias P., Demiridi E., Vogiatzis K., Skabardonis A., Zafiropoulou V. (2020). Connected & Autonomous Vehicles–Environmental Impacts—A Review. Sci. Total Environ..

[104] Shepherd S.P. (2014). A Review of System Dynamics Models Applied in Transportation. Transp. B Transp. Dyn..

[105] Sterman J. (2002). System Dynamics: Systems Thinking and Modeling for a Complex World.

[106] Stepp M.D., Winebrake J.J., Hawker J.S., Skerlos S.J. (2009). Greenhouse Gas Mitigation Policies and the Transportation Sector: The Role of Feedback Effects on Policy Effectiveness. Energy Policy.

[107] Andrea P., Conor H., Veronica P. (2014). The Influence of Transport on GHG Emissionin BrisbaneT.

[108] Boll C. The Impact of COVID-19 on Adoption of Autonomous Vehicle Technology; 2020. https://www.foley.com/en/insights/publications/2020/08/covid-19-adoption-autonomous-vehicle-technology.

